# Postsynthetic Degradation of Toxic Quantum Dots via Oleic Acid Complexation

**DOI:** 10.1021/acsomega.5c08351

**Published:** 2026-01-30

**Authors:** Elena Cambiotti, Emiliano Fratini, Loredana Latterini

**Affiliations:** † Nano4Light Lab, DCBB, 9309 Università di Perugia, Via Elce di Sotto, 8, Perugia 06123, Italy; ‡ 9300 Department of Chemistry “Ugo Schiff”, Via della Lastruccia 3, Sesto Fiorentino 50019, Italy; § Consorzio per Lo Sviluppo Dei Sistemi a Grande Interfase (CSGI), Via della Lastruccia 3, Sesto Fiorentino 50019, Italy

## Abstract

Quantum-confined semiconductor nanocrystals (NCs) have found extensive applications in optoelectronics. However, their inherent toxicity, often due to the incorporation of heavy and toxic metals, poses significant environmental and health concerns. In this study, we introduce a postsynthesis degradation strategy for CdSe quantum dots (QDs) based on the use of oleic acid (OA), a long-chain fatty acid capable of complexing with heavy metals. The degradation of nanocrystals is monitored in situ using UV–vis spectroscopy, small-angle X-ray scattering (SAXS), and transmission electron microscopy (TEM). The results revealed that OA induces progressive degradation of the QDs, resulting in a reduction in their size and ultimately leading to the complete dissolution of the QDs within 24 h. The proposed mechanism has been validated for other NC systems, including PbS QDs and CdTe nanorods, highlighting its broad applicability across diverse materials. This strategy offers a sustainable and scalable route to mitigate the environmental risks associated with NC discharge.

## Graphical abstract

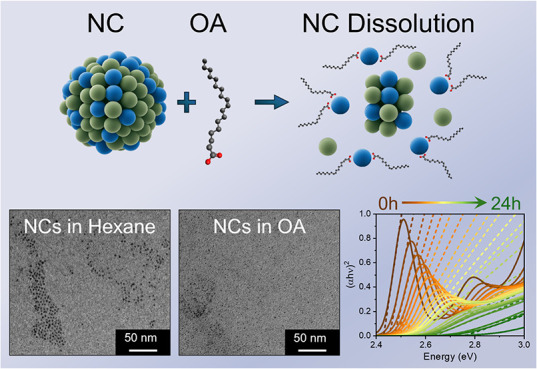

## Introduction

The observation and rationalization of size-dependent optical properties in quantum-confined semiconductor nanocrystals (NCs) by Brus and colleagues marked a significant advancement in nanoscience when coupled to mastering their preparation procedures.^1,2^ This breakthrough led to the understanding of quantum confinement effects, where excitons (i.e., the quasiparticles constituted by a hole–electron couple) are confined to a region comparable to or smaller than the Bohr radius.^3–6^ Quantum-confined nanocrystals are classified by the number of restricted spatial dimensions: two-dimensional materials confine electrons along one axis, one-dimensional ones along two axes, and zero-dimensional ones along all three axes. Examples of these materials include nanoplatelets (NPLs), nanorods (NRs), and quantum dots (QDs), respectively.^5,6^ Over the past years, NCs have found widespread applications in optoelectronic devices such as light-emitting diodes, photovoltaics, lasers, and in biological and medical technologies.^2,7–9^ Despite their remarkable properties, including size-tunable emission and high photoluminescence quantum yield, NCs face significant challenges due to the toxicity of heavy metals, such as Cd, Pb, and Hg, commonly incorporated into their structure. Heavy metal-based NCs are widely used in modern devices, but their end-of-life disposal poses significant environmental risks. Various strategies to mitigate the toxicity of NCs primarily focus on synthetic adjustments, including tailoring their morphology, modifying their composition, and applying biocompatible shell coatings.^10,11^ In contrast, postsynthetic strategies that address the degradation or complete destruction of NCs to mitigate their toxicity are far less developed. Existing studies have documented partial dissolution of NCs as a phenomenon observed under light exposure (photodegradation) or treatment with harsh reagents (chemical etching).^12–15^ However, to the best of our knowledge, no deliberate methods have been proposed to leverage NC degradation by exploiting biocompatible ligands. In this regard, we investigated how oleic acid (OA), a natural fatty acid known for its ability to form stable complexes with heavy metal ions through its carboxylic end groups, actively induces the degradation of NCs.^16,17^ OA was chosen as a mild degradation agent to enable controlled dissolution of the NCs and precise size tuning by adjusting the OA concentration. Furthermore, the stable heavy-metal oleate complexes formed during this process could be recovered and reused as precursors for new NC syntheses, supporting a sustainable approach. In this study, the degradation of NCs upon exposure to excess OA is examined via UV–vis spectroscopy, small-angle X-ray scattering (SAXS), and transmission electron microscopy (TEM). The findings confirm the effective degradation of heavy metal-based NCs across various shapes and compositions and, with optimization, could provide a novel strategy for tuning NC dimensions with controlled amounts of OA. This approach provides a simple and cost-effective strategy for the complete degradation of NCs with the potential to reduce the environmental impact of nanomaterials and promote sustainable practices in nanotechnology.

## Experimental Section

### Materials

Sodium myristate, cadmium nitrate tetrahydrate, selenium, oleic acid (OA), octadecene (ODE), lead­(II) nitrate, tellurium, cadmium oxide, sodium hydroxide, hexamethyldisilathiane, trioctylphosphine (TOP), methanol, hexane, toluene, and acetone were purchased from Sigma-Aldrich. All chemicals were used without further purification.

### Sample Preparation

The procedures to prepare the precursors and the semiconductor nanocrystals are presented in detail in the Supporting Information file.

### Methods

UV–visible absorption spectra were recorded by using a Shimadzu UV-1900i spectrophotometer. Steady-state and time-resolved photoluminescence measurements were carried out by an Edinburgh FS5 spectrophotometer equipped with a Xe lamp for steady-state measurements and a pulsed diode source for time-resolved studies. FT-IR spectra of pure oleic acid and QD-dissolved oleic acid were collected by using a Shimadzu IRXross FT-IR spectrometer operating in transmission mode. Spectra were accumulated over 45 scans from 4000 to 350 cm^–1^ with a resolution of 2 cm^–1^. The refractive index was measured at 20 °C with a Schmidt + Haensch VariRef refractometer. Transmission electron microscopy (TEM) images were obtained through a Philips transmission electron microscope (model 208) operating at 80 kV of beam acceleration. For sample preparation, a suspension of CdSe QDs was deposited onto a 300-mesh carbon-supported copper grid. The solvent (hexane) was evaporated overnight in a desiccator, and excess oleic acid was removed prior to imaging. SAXS analyses were performed on a Xeuss 3.0 HR instrument (Xenocs, Grenoble) equipped with a high-brightness X-ray tube, a FOX 3D single reflection multilayer optic, and a Dectris Eiger 2R 1 M hybrid photon counting detector with a pixel size of 75 × 75 μm^2^. The X-ray beam was derived from Cu Kα radiation (λ = 1.542 Å) emitted by a microfocus tube working at full power (30 W). The sample-to-detector distance was set to 300 mm. At first, 2D SAXS images were collected; 1D data, expressed as Intensity vs the scattering vector, *q* (where *q* = (4π/λ) sinθ and θ is half of the scattering angle) were obtained by circularly averaging 2D images. Data correction was performed by empty holder subtraction. All data reduction was performed using XSACT software (Xenocs, Grenoble). Measurements were conducted in an air-equilibrated environment at room temperature by using a capillary holder. The procedures used to standardize the measurements and to analyze the data are reported in the Supporting Information file. Absorption, steady-state and time-resolved PL, and SAXS measurements were performed using a nanocrystal concentration of 1 × 10^–6^ M.

## Results and Discussion

CdSe QDs have been synthesized via the hot injection method, using oleic acid as a stabilizer with a molar fraction of Cd:OA of roughly 1:9. After purification with acetone, the QDs are redispersed in hexane and optically characterized. The normalized absorption spectrum in Figure 1a displays a prominent excitonic band centered at 499 nm. The band gap energy of the synthesized QDs is estimated to be 2.41 eV using the Tauc plot (inset Figure 1a). The emission PL spectrum of the synthesized QDs has a narrow band peaked at 510 nm, which is assigned to band-edge emission of the QDs due to the band profile and the small Stokes shift with respect to the absorption band. Along with the narrow PL band, an additional broad band at a higher wavelength originates from trap states.^18,19^ These surface states of QDs have been attributed to defects such as vacancies, local lattice mismatches, dangling bonds, or adsorbates (capping ligands) on the surface, in agreement with literature.^20^ Photoluminescence quantum yield (PLQY) of the synthesized CdSe QDs is determined to be 20.2% using a relative method, as detailed in the Supporting Information. The QD stability in hexane dispersion is evaluated after 3 days. The results summarized in Figure S1 confirm that the QDs retained their optical properties, indicating good stability. The QDs’ PL decays are collected at the band-edge emission wavelength (510 nm) and at the emission wavelength of the trap states (725 nm). Figure 1b shows the luminescence decays and highlights that the red contribution is due to defect-mediated states, which trap the photogenerated excitons, lengthening their deactivation since a longer average decay time (479 ns, gray line) is measured compared to the almost four times faster band-edge luminescence decay (136 ns, black line).^21,22^

**Figure 1:**
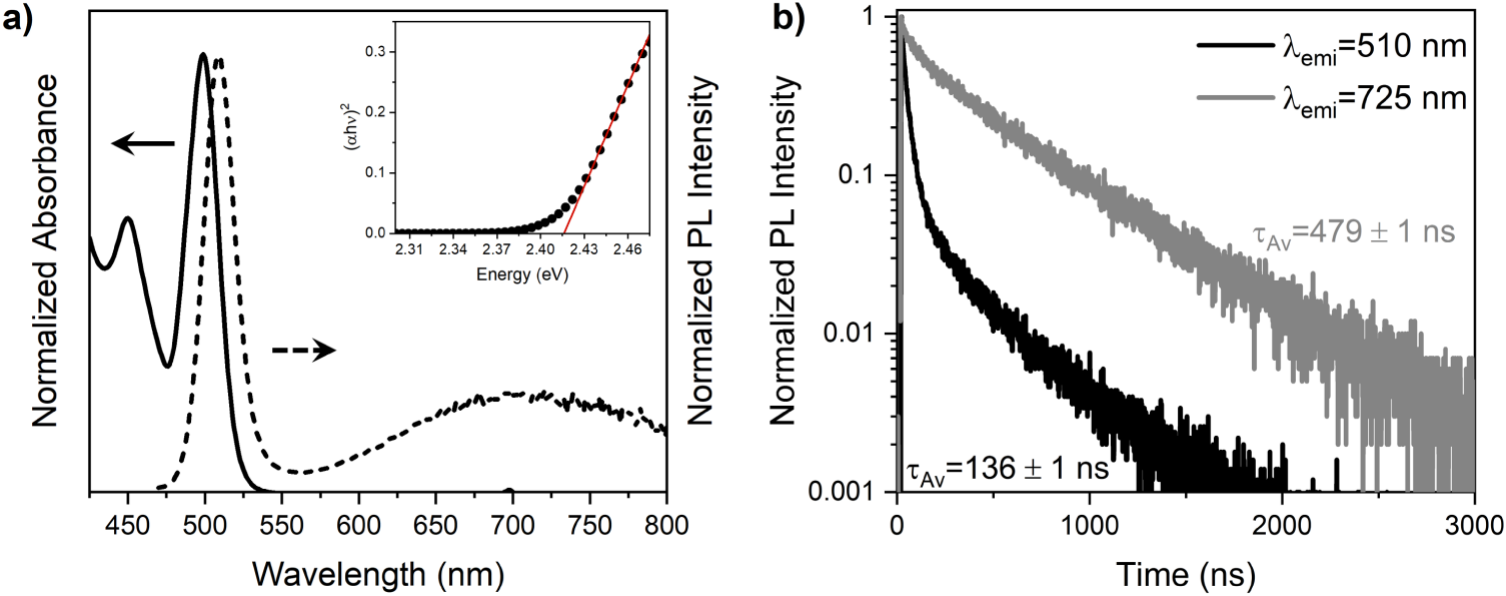
Normalized absorption (solid line) and steady-state PL (dashed line) spectra of CdSe QDs in hexane (a). The inset in (a) reports the Tauc Plot for QDs. Time-resolved PL decays of QDs at 510 and 725 nm collected under 450 nm excitation (b).

OA is commonly used in QD synthesis as a capping ligand to enhance colloidal stability.^23,24^ However, even slight variations in the OA concentration can influence the physicochemical properties of QDs. To examine these effects after synthesis, SAXS measurements are performed on QDs dispersed in hexane, both in the absence and presence of increasing OA concentrations. Data have been collected at room temperature and averaged over a 24 h period to ensure equilibration of the dispersion. The intensity profile derived from SAXS measurements is normalized with respect to QD concentration and plotted against the scattering vector *q* (Å^–1^) in Figure 2. Previous studies by Bawendi and colleagues have shown that this type of QD is more accurately described as ellipsoids rather than spheres.^25,26^ Accordingly, the SAXS profile of the CdSe QDs dispersed in hexane (black curve) is fitted by using an ellipsoidal model (see SI for the detailed analytical description), yielding a polar radius of 1.2 nm and an equatorial radius of 1.9 nm. These values are in good agreement with a QD diameter of ∼2.5 nm estimated using the Nguyen methodology, which assumes a spherical nanocrystal.^27^ The introduction of OA at a hexane-to-OA ratio of 1:1.5 did not cause any significant alterations in the scattering curve, indicating that the QDs maintained their properties under this condition. However, increasing the OA concentration to achieve a hexane-to-OA ratio of 1:3 resulted in a pronounced shift in the scattering curve toward higher *q* values. This increase in the relative OA/QD concentration results in a reduction of the QD dimensions to a polar radius of 0.8 nm and an equatorial radius of 1.1 nm, which corresponds to a shrinking of 33% and 42% in the polar and equatorial directions, respectively. All measurements reported in this study are performed at a QD concentration of 1 × 10^–6^ M. At this concentration, a minimum hexane-to-OA ratio of 1:3 is required to induce measurable size modifications in the QDs. Based on these results, subsequent experiments are conducted by drastically increasing the OA concentration, fully substituting hexane with OA, to have OA acting as both ligand and solvent.

**Figure 2:**
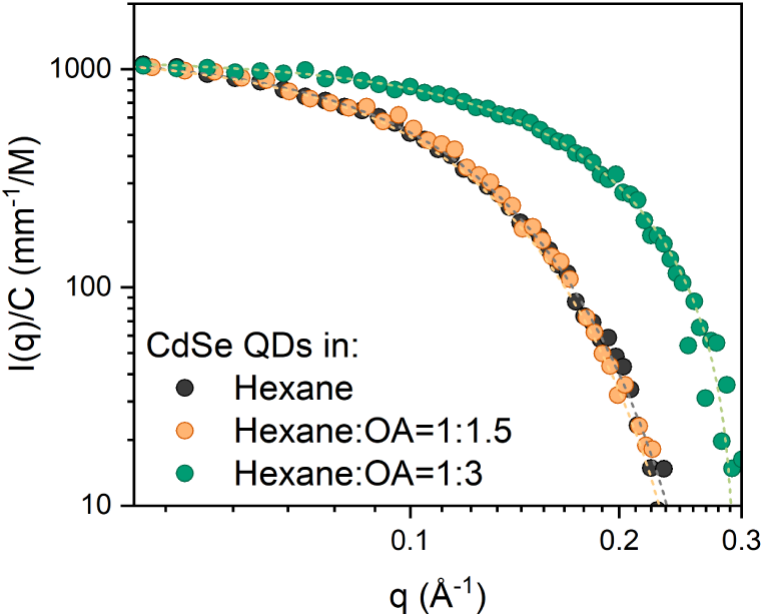
SAXS curves normalized for the QDs concentration (1 × 10^–6^ M CdSe QDs) in hexane before and after the addition of different amounts of OA. The resulting errors are comparable to the oscillations observed in the data points.

The QDs in OA as a solvent are monitored over time via SAXS and the results are illustrated in Figure 3. The black curve represents the SAXS profile of CdSe QDs in hexane, exhibiting the highest scattering intensity across the *q* range. Then, the as-synthesized QDs are dispersed in OA and analyzed, resulting in a slight shift toward higher *q* values and a decrease in scattering intensity compared to the hexane sample. This suggests an initial interaction between the QDs and OA, leading to a size reduction of the QDs. As time progressed, the pronounced reduction in intensity, along with a further shift toward higher *q* values, suggests a continuous dissolution of the QDs and a further reduction of their dimension. After 24 h, SAXS measurement showed no detectable QD signal due to a complete dissolution of QDs by OA complexation. The fitting results for each SAXS curve (Table 1) are used to calculate the QD volume (see SI for more details). Exposure of QDs to an excess of OA resulted in a volume reduction of 53% within 0.5 h, increasing to a 91% reduction after 10.5 h and culminating in complete dissolution after 24 h.

**Figure 3:**
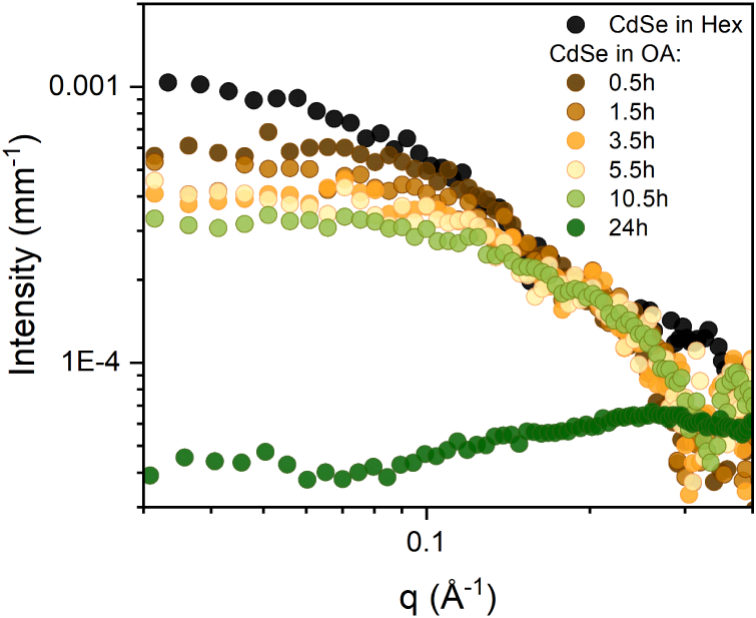
SAXS curves of CdSe QDs and their subsequent dispersion in OA were monitored over time.

**Table 1:** Size Parameters of QDs Obtained from the Analysis of SAXS Profiles

QDs Solvent	Time of Dispersion (h)	Polar Radius (nm)	Equatorial Radius (nm)	QD Volume (nm^3^)	Volume Reduction
*Hexane*	-	1.2	1.9	18.1	-
*Oleic Acid*	0.5	0.9	1.5	8.5	53%
*Oleic Acid*	1.5	0.8	1.3	5.7	69%
*Oleic Acid*	3.5	0.7	1.2	4.2	77%
*Oleic Acid*	5.5	0.6	1.2	3.6	80%
*Oleic Acid*	10.5	0.4	1.0	1.7	91%

To further investigate the dissolution hypothesis, the absorption spectrum of the QDs in OA has been monitored over time at room temperature. Figure 4a shows the absorption spectra of the OA-capped QDs in oleic acid over a period of 24 h. Over the course of the dissolution kinetics, the QD excitonic peak shifts to the blue region, suggesting a decrease in the QD diameter along with a reduction of absorbance until the band structure is completely lost. Figure 4b highlights the integrated absorbance decrease of QDs exposed to OA as a function of time. The dissolution kinetics follow a second-order model with a rate of 1.6 × 10^–4^ M^–1^s^–1^ (Figure 4c), suggesting a bimolecular dissolution where two molecules of OA interact with the CdSe surface, extracting Cd^2+^ and Se^2–^. This finding is in agreement with the literature, where the significant reduction in QD size induced by OA is likely due to the extraction of cadmium from the QD lattice, potentially leading to the formation of cadmium oleate.^13^ Consistent with this mechanistic picture, the FT-IR spectra discussed in Figure S2 indicate the partial conversion of oleic acid to a metal carboxylate. In addition, the refractive index increases from pure OA (1.46197) to the QD-dissolved OA (1.46949), providing complementary evidence for the formation of Cd-oleate complexes. The Tauc plot analysis has been performed for each absorption spectrum of the dissolution kinetics in OA (inset Figure S3), revealing a band gap shift from 2.43 to 2.75 eV after exposure to OA for 390 min. After 24 h, the absence of a distinguishable excitonic peak prevented the estimation of the band gap energy, confirming complete QD dissolution. The mean diameter of the QD is calculated during the dissolution time through the Nguyen equation (see SI) and summarized in Figure S3 and Table S1. The QD size is found to decrease from 2.46 to 1.95 nm over 390 min, in good agreement with SAXS data.

**Figure 4:**
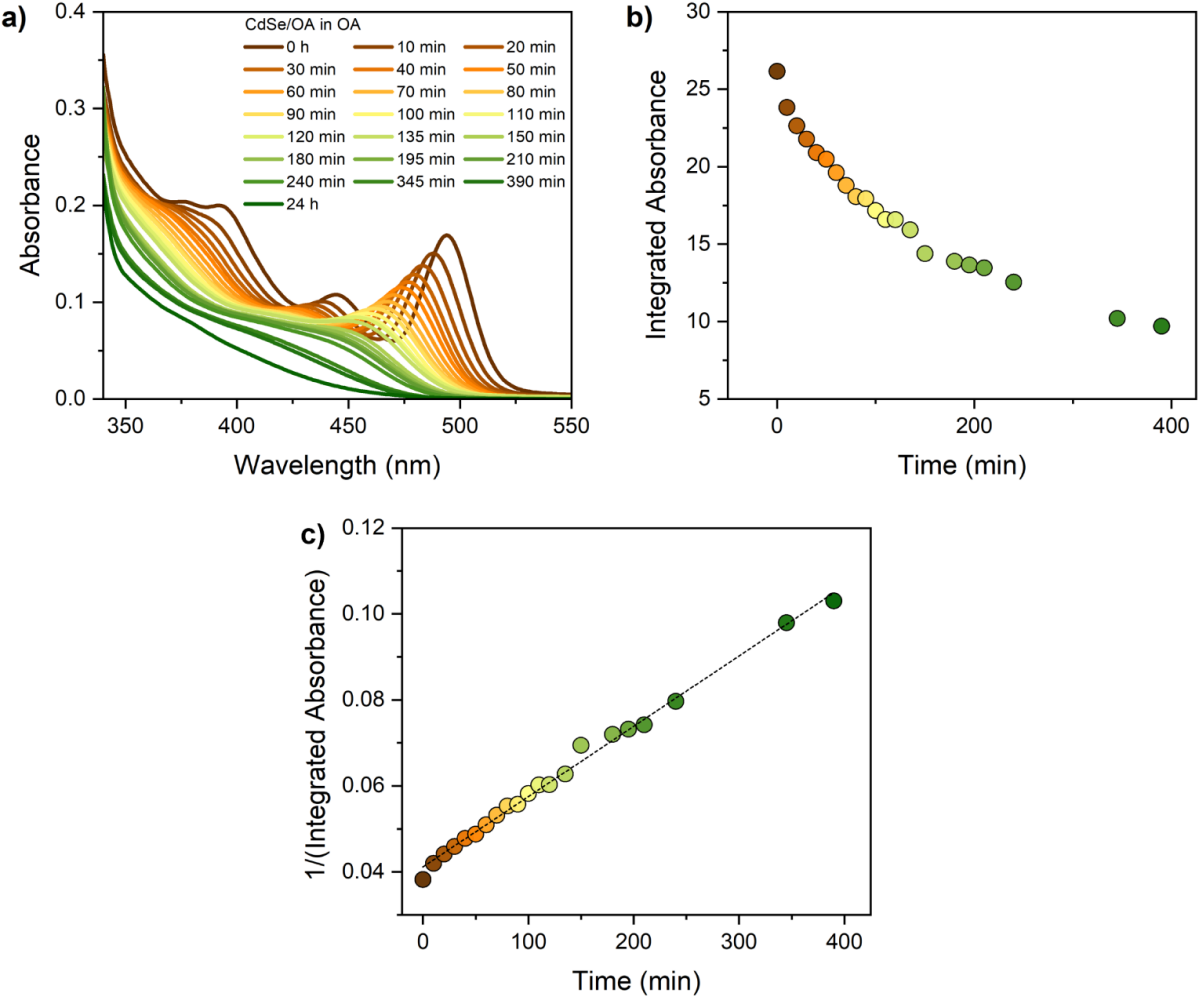
Absorption spectra of CdSe QDs in OA over time (a) and dissolution plot (b). Second-order kinetics for the dissolution of CdSe QDs in OA (c).

A morphological study has been carried out on the QDs dispersed in hexane and OA. Through TEM images of QDs in hexane, a diameter of approximately 2.5 nm could be estimated (Figure S4a), which is in reasonable agreement with SAXS data. However, when OA is used as a solvent, a noticeable decrease in the QD size is evident (Figure S4b), consistent with the previous results. This study confirms that an excess of OA irreversibly alters QD size.^13,28^ The primary degradation byproduct, cadmium oleate, forms a stable coordination complex with reduced bioavailability relative to free Cd^2+^, suggesting lower environmental toxicity; however, its removal or immobilization remains important to prevent secondary contamination.^29^

We performed ligand exchanges to compare OA, trioctylphosphine oxide (TOPO), oleylamine, and dodecanethiol, which differ in their functional group chemistry. The absorption spectra after 3 days in OA show that QDs capped with TOPO or oleylamine behave similarly to OA-capped QDs, exhibiting complete degradation (Figure 5). In contrast, dodecanethiol provides significant stabilization, preserving the absorption features with only minor shifts in the peak position. This trend aligns with established ligand classifications. TOPO and oleylamine are L-type ligands that bind weakly and easily desorb, whereas OA and alkyl thiols are X-type ligands that form stronger covalent bonds to the QD surface.^13,30^ OA also acts as a strong Lewis base capable of coordinating with surface metal sites, promoting dissolution. Dodecanethiol, therefore, offers the most robust protection against OA-induced degradation.^31^

**Figure 5:**
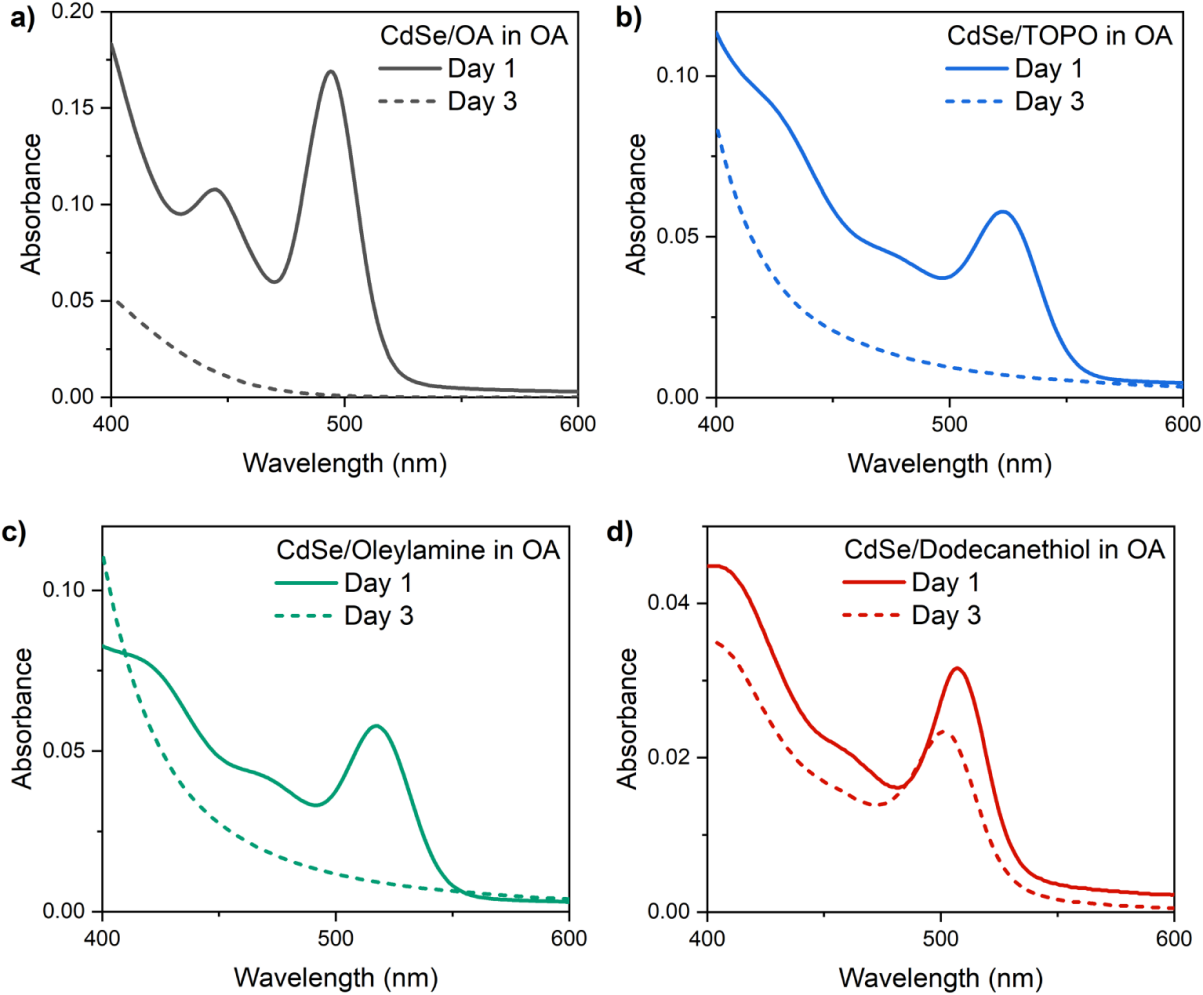
Absorption spectra of CdSe QDs in OA with OA as ligand (a), after ligand exchange with TOPO (b), oleylamine (c), and dodecanethiol (d). Spectra collected right after the dispersion of QDs in OA (solid line) and after 3 days (dashed line).

The effect of OA on quantum-confined semiconductor nanocrystals has also been investigated in systems with different composition (PbS QDs) and shape (CdTe NRs) using UV–vis absorption spectroscopy, selected for real-time monitoring. The NCs have been synthesized in organic solvents; their absorption spectra (Figure 6a and b) show the excitonic band centered at 825 and 480 nm, respectively, resulting in band gap values of 1.50 eV for PbS QDs and 2.58 eV for CdTe NRs. Both PbS QDs and CdTe NRs exhibit emission maxima at 925 nm (1.34 eV) for PbS QDs and at 573 nm (2.16 eV) for CdTe NRs. The prepared NCs have been exposed to an excess of OA and their absorption spectra are collected over time. Figure 6c reveals a blue shift in the excitonic peak for PbS QDs, indicative of a decrease in the particle size. For CdTe NRs (Figure 6d), this shift initially increases the absorbance during the first hour. However, prolonged exposure to OA leads to the complete disappearance of the excitonic features in both systems.

**Figure 6:**
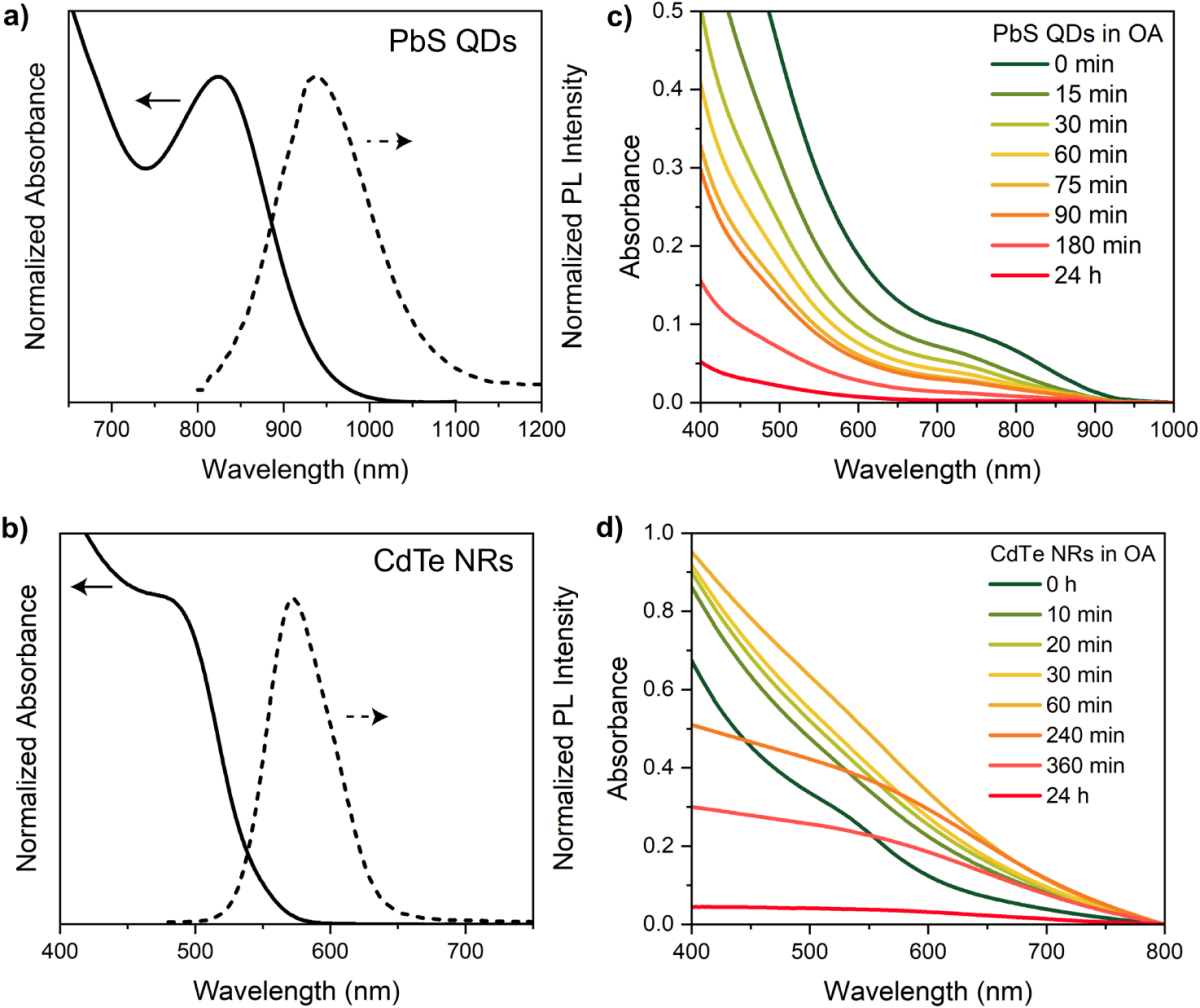
Normalized absorption (continuous line) and steady-state PL (dashed line) spectra of PbS QDs in toluene (a) and CdTe NRs in hexane (b). Time-dependent absorption spectra in OA for PbS QDs (c) and CdTe NRs (d).

The dissolution of QDs in OA is further demonstrated in Figure S5, where neat OA is compared with dispersions of CdSe and PbS QDs. Initially, the dispersions appear yellowish, indicative of the presence of QDs. After 3 days in OA, the dispersions become colorless, confirming the loss of QD characteristics as confirmed by UV–vis, SAXS, and TEM measurements. This study establishes oleic acid complexation as an effective postsynthetic approach for precise tuning of nanocrystal size to complete dissolution, upon adjusting the OA volume fraction. The proposed strategy shows its applicability across diverse NC compositions and morphologies, broadening its impact on nanomaterial design and application. Additionally, this study for the first time proposes a methodology grounded on a biobased molecule to redisperse NCs after their use, which might pave the way to solve environmental concerns raised from dismantling of heavy metal-based devices.

## Conclusion

This study provides an in-depth investigation into the dissolution of CdSe QDs in OA. SAXS analysis revealed initial structural changes upon the addition of small amounts of OA, followed by a complete degradation of QDs within 24 h in saturated OA solutions. This result is confirmed by UV–vis spectroscopy, which monitored the temporal evolution of the absorption profile of the CdSe QDs, showing a progressive loss of optical properties and a pronounced blue shift in their spectral features. TEM further corroborated these observations by demonstrating a reduction in QD dimensions upon dispersion in OA. The results support the hypothesis that oleic acid facilitates the complexation and extraction of heavy metal ions from the QD structure, resulting in destabilization and dissolution of the lattice. The degradation strategy is successfully extended to quantum-confined nanocrystals with diverse compositions and morphologies, including PbS QDs and CdTe nanorods, to highlight the versatility of the proposed approach. The proposed method underscores the potential of oleic acid as a versatile and sustainable agent for controlled postsynthetic modification and dissolution of NCs, offering a pathway to mitigate environmental concerns arising from dismantling of heavy metal-based devices. Nevertheless, the current method requires relatively high OA concentrations and dissolution times of approximately 24 h; future work will aim to optimize OA content to accelerate the dissolution process even in the presence of different ligands and to extend the strategy to more complex solid-state device architectures, thus enhancing its practical applicability.

## Supplementary Material



## Notes

Supporting Information is available free of charges: experimental procedures, descriptions of the SAXS fitting model, photoluminescence quantum yield calculations, band gap and particle size determinations; Absorption and emission spectra of samples, FT-IR spectra of pure OA and dissolved QDs in OA, Tauc plot, TEM images, photographs of samples before and after aging in oleic acid, Table summarizing the band gap energy and particle size evolution during dissolution (PDF)
