# Supplementary material for: Postsynthetic Degradation of Toxic Quantum Dots via Oleic Acid Complexation
Source: ACS Omega. 2026 Jan 30;11(5):7426–32. doi: 10.1021/acsomega.5c08351 (PMC12903163; doi:10.1021/acsomega.5c08351)
Supplement: Supplementary file 1 [file ao5c08351_si_001.pdf]

Post-Synthetic Degradation of Toxic Quantum Dots via Oleic Acid Complexation

Elena Cambiotti[a,b,c], Emiliano Fratini[b,c], Loredana Latterini[a,c*]

[a] Nano4Light Lab, DCBB, Università di Perugia, Via Elce di Sotto, 8, 06123 Perugia, Italy

[b] Department of Chemistry "Ugo Schiff", Via della Lastruccia 3, 50019, Sesto Fiorentino, Italy

[c] Consorzio per lo Sviluppo dei Sistemi a Grande Interfase (CSGI), Via della Lastruccia 3, Sesto Fiorentino 50019, Italy

* corresponding: Loredana Latterini – loredana.latterini@unipg.it

- Sample preparation procedures page 2

- SAXS Fitting Model page 3

- Photoluminescence quantum yield determination page 4

- Band gap and size determination page 4

- Figures and Tables page 5

Sample preparation

CdSe QDs Preparation

The cadmium myristate $Cd(myr)_2$ precursor was prepared according to Bertrand and coworkers.[1] 5 g of sodium myristate was dissolved in 250 mL of methanol while vigorously stirring. In a second flask, 3 g of cadmium nitrate tetrahydrate was dissolved in 500 mL of methanol. The resulting cadmium containing solution was then added dropwise to the dissolved sodium myristate solution and stirred at room temperature for 2 h. The synthesized $Cd(myr)_2$ was filtered, washed three times with methanol, and dried under vacuum. The CdSe QDs were then synthesized with slight modifications from literature.[2,3] Briefly, 192 mg of $Cd(myr)_2$ was added to 15 mL of ODE and degassed under vacuum for 1 h at 120°C. The mixture was then heated under a nitrogen flow at 240 °C and 24 mg of Se in 1 mL of ODE was injected into the solution. Following the injection, the temperature was held at 240 °C for 3 minutes, then the solution was allowed to cool. Once the temperature reached 150 °C, 1 mL of OA was injected to stabilize the QDs surface. The resulting solution was allowed to cool to room temperature where it was then transferred to a glovebox and centrifuged at 6000 rpm for 15 min. The supernatant was discarded, the resulting pellet was redispersed in 2 mL of hexanes, followed by filtering to remove the unreacted precursors.

PbS QDs Preparation

Before going into the synthesis of the PbS quantum dots, the precursor preparation is briefly described. Lead oleate ($Pb(oleate)_2$) was prepared starting from lead nitrate as shown by Hendricks and coworkers with slight modification.[4] 1.825 g of sodium hydroxide was dissolved in 1 L of methanol under vigorous stirring. Once dissolved, 12.710 g of OA was slowly added to the solution, followed by the addition of 6.630 g of lead (II) oleate in 300 mL of methanol. The mixture was heated and stirred until clear; then the heat and stirring were turned off. The obtained solution was cooled down and filtered to remove any insoluble precursors. The solution was stored overnight at 5 °C to promote flocculation. The resulting white powder was purified via filtration, washed three times with methanol, and then dried under vacuum. The preparation of the PbS QDs is summarized below, which followed a modified version of the hot-injection method presented by Hines and Scholes.[5] 1 g of the previous synthesized $Pb(oleate)_2$ was placed in a three-neck flask with 10 mL of octadecene; the precursor was degassed under vacuum for 12 hours at 120 °C. A 0.135 mL solution of hexamethyldisilathiane in 2 mL of ODE, a non-coordinating solvent, was then rapidly injected under an inert nitrogen environment at 90 °C, and the reaction was immediately quenched by an ice bath. The obtained nanocrystal dispersion was then transferred into a glovebox, purified from the growth

mixture by precipitation with a polar solvent (acetone), and subsequently redispersed in a non-polar solvent (toluene). Precipitation and redispersion procedures were repeated twice to ensure the removal of remaining unwanted precursors. The resulting pellet was then redispersed in toluene and stored in a nitrogen filled glovebox.

CdTe NRs Preparation

The CdTe nanorods were synthesized according to the method proposed by VanOrman and coworkers.[6] The Te-TOP precursor was prepared by dissolving 36 mg of Te powder in 3 mL of TOP. The mixture was heated at 35°C and stirred for 1 hour. In a 25 mL three-neck flask, 120 mg of CdO was combined with 1.33 mL of OA and 15 mL of ODE. The mixture was degassed under vacuum at 120 °C for 1 hour, followed by a nitrogen purge. The temperature was raised to 200°C until the mixture became clear, after which 3 mL of the Te-TOP solution was injected rapidly. The reaction was maintained at 200 °C for 5 minutes and then allowed to cool to room temperature. The final NRs were purified by centrifugation, redispersed in hexane and filtered to remove unreacted precursors.

Methods

SAXS analyses were performed on a Xeuss 3.0 HR instrument (Xenocs, Grenoble) equipped with a high brightness X-ray tube, a FOX 3D single reflection multilayer optic, and a Dectris Eiger 2R 1M hybrid photon counting detector with a pixel size of 75×75 μm^2. The X-ray beam was derived from Cu Kα radiation ($\lambda = 1.542$ Å) emitted by a micro-focus tube working at full power (30 W). The sample-to-detector distance was set to 300 mm. At first, 2D SAXS images were collected; 1D data, expressed as Intensity vs. the scattering vector, q (where $q=(4\pi/\lambda)\ sin\theta$ and θ is half of the scattering angle) were obtained by circularly averaging 2D images. Data correction was performed by empty holder subtraction. All data reduction was performed through XSACT software (Xenocs, Grenoble). Measurements were conducted in an air-equilibrated environment at room temperature using a capillary holder. The sample-to-detector distance was calibrated using silver behenate as a standard.[7] The intensity was then converted to absolute scale, using the scattering profile of a calibrated Glassy carbon acquired in the same experimental conditions and knowing the sample thickness.[8] SAXS profiles were fitted using the SasView 5.0 software (http://www.sasview.org/), employing an ellipsoidal model with a 0.2 polydispersity.[9]

SAXS Fitting Model

The ellipsoid model used to fit all the SAXS profile is given by Feigin[9] according to the following equation:

$$P(q,\alpha) = \frac{scale}{V} F^2(q,\alpha) + background \qquad \text{(eq.S1)}$$

where

$$F(q,\alpha) = \Delta\rho V \frac{3(\sin qr - \mathrm{qr}\cos qr)}{(qr)^3} \qquad \text{(eq.S2)}$$

for

$$r = [R_e^2\, sin^2\,\alpha + R_p^2\, cos^2\,\alpha]^{1/2} \qquad \text{(eq.S3)}$$

α is the angle between the axis of the ellipsoid and q, $V = \left(\frac{4}{3}\right)\pi R_p R_e^2$ is the volume of the ellipsoid, R_p is the polar radius along the rotational axis of the ellipsoid, R_e is the equatorial radius perpendicular to the rotational axis of the ellipsoid and Δρ is the scattering length density difference between the scatterer and the solvent.

Photoluminescence Quantum Yield

The photoluminescence quantum yield (Φ) was calculated according to equation 4, where Φ_{Std} represents the QY of the standard (rhodamine 6G), A_{Std} is the absorbance of the standard, I_{Std} is the integrated photoluminescent intensity and n_{Std} is the refractive index of the standard. The same corresponding terms for the QDs are denoted by the subscript QD.

$$\Phi = \Phi_{\mathrm{Std}} \cdot \frac{\mathrm{A_{Std}}}{\mathrm{A_{QD}}} \cdot \frac{\mathrm{I_{QD}}}{\mathrm{I_{Std}}} \cdot \left(\frac{\mathrm{n_{QD}}}{\mathrm{n_{Std}}}\right)^2 \qquad \text{(eq. S4)}$$

Band gap and size determination

The band-gap value was calculated from the absorption spectra using Tauc relation[10,11] expressed with equation 5:

$$(\alpha h\nu) = B(h\nu - E_g)^r \qquad \text{(eq. S5)}$$

where α is the absorption coefficient, $h\nu$ is the photon energy, B is a constant, E_g is the optical band gap and r is an index that assumes the values 1/2, 2, 3/2 and 3 for allowed direct, allowed indirect, forbidden direct and forbidden indirect transitions, respectively. Hence, for direct transitions eq. 5 becomes:

$$(\alpha h\nu) = B(h\nu - E_g)^{1/2} \qquad \text{(eq. S6)}$$

The Tauc representation shows the variation of $(\alpha h\nu)^2$ versus $(h\nu)$. The band-gap energy was obtained by extrapolating the linear region to the energy axis where $(\alpha h\nu)^2 = 0$.

The average diameter of CdSe nanoparticles can be estimated according to the following equation:[12]

$$D = (1.6122 \cdot 10^{-9})\lambda^4 - (2.6575 \cdot 10^{-6})\lambda^3 + (1.6242 \cdot 10^{-3})\lambda^2 - (0.4277)\lambda + 41.57$$

(eq.7S)

Where D is the is the average diameter of the CdSe QDs and λ is the wavelength of the absorption peak obtained by Tauc's plot.

Figures and Tables

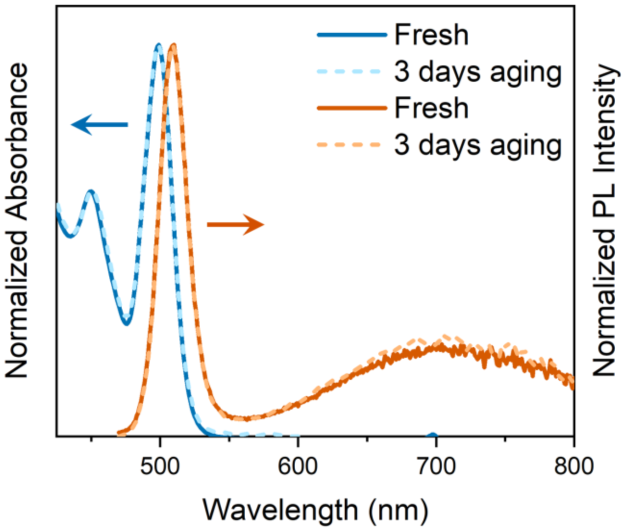

Figure S1. Normalized absorption (blue) and steady-state PL (orange) spectra of CdSe QDs in hexane immediately after synthesis (solid lines) and after a 3-day aging time (dashed lines).

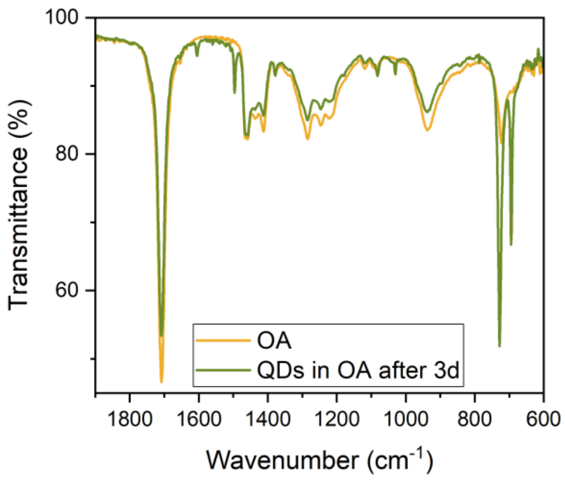

Figure S2. FT-IR spectra of pure OA (yellow) and dissolved QDs in OA (green). The decreased intensity of the C=O stretching band at 1707 cm^{-1} for the QD-dissolved OA supports the partial conversion of carboxylic acid to carboxylate. Moreover, asymmetric COO^{-} vibrational mode appears at 1604 cm^{-1} and the symmetric COO^{-} vibrational mode appears at 1495 cm^{-1}, indicating the formation of Cd-bound carboxylate. Subtracting these values yields $\Delta\nu = 109$ cm^{-1}, which is associated with an asymmetric chelated bidentate binding mode typical of Cd-oleate complexes.[13,14]

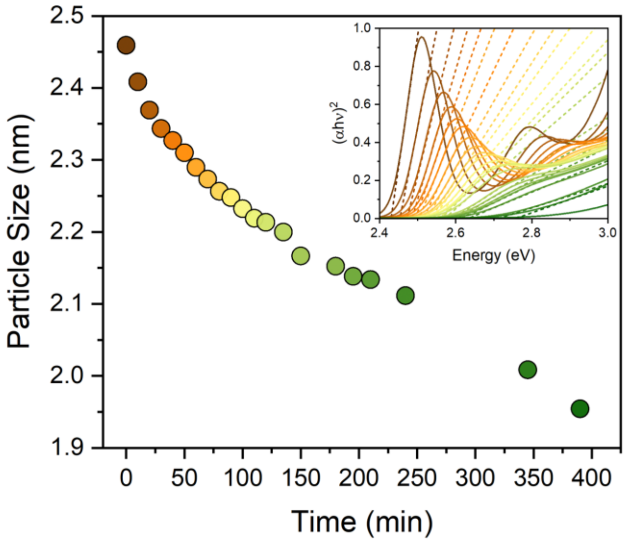

Figure S3. Tauc Plot (inset) and particle size as a function of time.

Table S1. Band-gap energy (E_g) and particle size of CdSe QD in OA over time.

OA exposure	E_g (eV)	Particle Size (nm)
**0 min**	2.43	2.46 ± 0.02
**10 min**	2.45	2.41 ± 0.02
**20 min**	2.47	2.37 ± 0.02
**30 min**	2.48	2.34 ± 0.02
**40 min**	2.49	2.33 ± 0.02
**50 min**	2.50	2.31 ± 0.02
**60 min**	2.51	2.29 ± 0.02
**70 min**	2.52	2.27 ± 0.02
**80 min**	2.53	2.26 ± 0.02
**90 min**	2.54	2.25 ± 0.02
**100 min**	2.54	2.23 ± 0.02
**110 min**	2.55	2.22 ± 0.02
**120 min**	2.56	2.21 ± 0.02
**135 min**	2.57	2.20 ± 0.02
**150 min**	2.59	2.17 ± 0.02
**180 min**	2.60	2.15 ± 0.02
**195 min**	2.61	2.14 ± 0.02
**210 min**	2.62	2.13 ± 0.02
**240 min**	2.63	2.11 ± 0.02
**345 min**	2.71	2.01 ± 0.02
**390 min**	2.75	1.95 ± 0.02

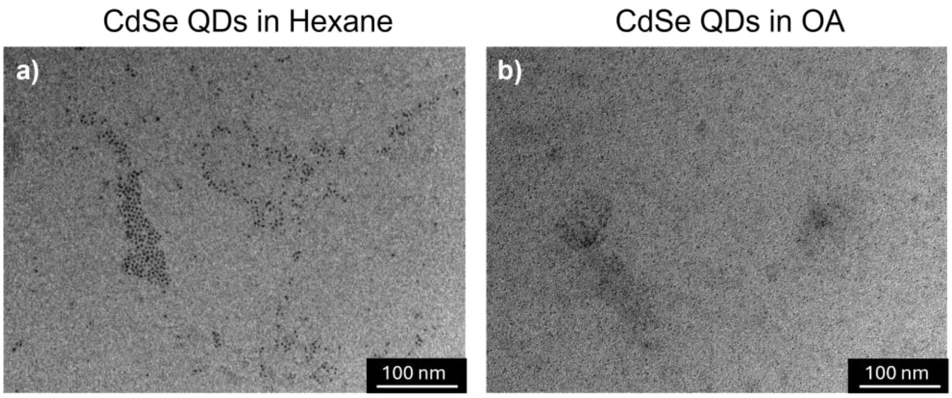

Figure S4. TEM images of CdSe/OA in hexane (a) and after 10 hour in OA (b) as solvent.

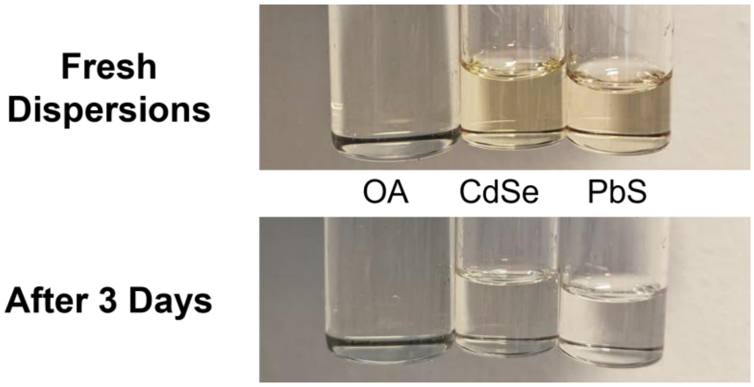

Figure S5. Image of pure OA, OA+CdSe QDs and OA+PbS QDs right after the preparation (top) and after 3 days (bottom).

References

(1) V. Bertrand, G. H.; Polovitsyn, A.; Christodoulou, S.; Hossain Khan, A.; Moreels, I. Shape Control of Zincblende CdSe Nanoplatelets. *Chem. Commun.* **2016**, *52* (80), 11975–11978. https://doi.org/10.1039/C6CC05705E.

(2) VanOrman, Z. A.; Bieber, A. S.; Wieghold, S.; Nienhaus, L. Green-to-Blue Triplet Fusion Upconversion Sensitized by Anisotropic CdSe Nanoplatelets. *Chem. Mater.* **2020**, *32* (11), 4734–4742. https://doi.org/10.1021/acs.chemmater.0c01354.

(3) Ithurria, S.; Tessier, M. D.; Mahler, B.; Lobo, R. P. S. M.; Dubertret, B.; Efros, A. L. Colloidal Nanoplatelets with Two-Dimensional Electronic Structure. *Nat. Mater.* **2011**, *10* (12), 936–941. https://doi.org/10.1038/nmat3145.

(4) Hendricks, M. P.; Campos, M. P.; Cleveland, G. T.; Jen-La Plante, I.; Owen, J. S. A Tunable Library of Substituted Thiourea Precursors to Metal Sulfide Nanocrystals. *Science* **2015**, *348* (6240), 1226–1230. https://doi.org/10.1126/science.aaa2951.
(5) Hines, M. a.; Scholes, G. d. Colloidal PbS Nanocrystals with Size-Tunable Near-Infrared Emission: Observation of Post-Synthesis Self-Narrowing of the Particle Size Distribution. *Adv. Mater.* **2003**, *15* (21), 1844–1849. https://doi.org/10.1002/adma.200305395.
(6) VanOrman, Z. A.; Conti, C. R. I.; Strouse, G. F.; Nienhaus, L. Red-to-Blue Photon Upconversion Enabled by One-Dimensional CdTe Nanorods. *Chem. Mater.* **2021**, *33* (1), 452–458. https://doi.org/10.1021/acs.chemmater.0c04468.
(7) Blanton, T. N.; Huang, T. C.; Toraya, H.; Hubbard, C. R.; Robie, S. B.; Louër, D.; Göbel, H. E.; Will, G.; Gilles, R.; Raftery, T. JCPDS—International Centre for Diffraction Data Round Robin Study of Silver Behenate. A Possible Low-Angle X-Ray Diffraction Calibration Standard. *Powder Diffr.* **1995**, *10* (2), 91–95. https://doi.org/10.1017/S0885715600014421.
(8) Zhang, F.; Ilavsky, J.; Long, G. G.; Quintana, J. P. G.; Allen, A. J.; Jemian, P. R. Glassy Carbon as an Absolute Intensity Calibration Standard for Small-Angle Scattering. *Metall. Mater. Trans. A* **2010**, *41* (5), 1151–1158. https://doi.org/10.1007/s11661-009-9950-x.
(9) Feigin, L. A.; Svergun, D. I. *Structure Analysis by Small-Angle X-Ray and Neutron Scattering*; Taylor, G. W., Ed.; Springer US: Boston, MA, 1987. https://doi.org/10.1007/978-1-4757-6624-0.
(10) Tauc, J. *Amorphous and Liquid Semiconductors*; Springer Science & Business Media, 2012.
(11) Al-Amri, A. M.; Yaghmour, S. J.; Mahmoud, W. E. Low Temperature Growth of Metastable Cubic CdSe Nanocrystals and Their Photoluminescence Properties. *J. Cryst. Growth* **2011**, *334* (1), 76–79. https://doi.org/10.1016/j.jcrysgro.2011.07.029.
(12) Nguyen, H. Q. Synthesis and Optical Properties of CdSe Nanocrystals and CdSe/ZnS Core/Shell Nanostructures in Non-Coordinating Solvents. *Adv. Nat. Sci. Nanosci. Nanotechnol.* **2010**, *1* (2), 025004. https://doi.org/10.1088/2043-6254/1/2/025004.
(13) Welsch, T. A.; Cleveland, J. M.; Thomas, J. A.; Schyns, Z. O. G.; Korley, L. T. J.; Doty, M. F. Supramolecular Gelation of Cadmium Oleate in the Synthesis of Nanocrystals for Applications in Photonics and Optoelectronics. *ACS Appl. Nano Mater.* **2024**, *7* (11), 13319–13327. https://doi.org/10.1021/acsanm.4c01839.
(14) Comparative Spectroscopic Analysis of Cadmium Bis(Isoundecanoate) and Its Alternatives. **2025**.
